# Time-Dependent Impairment of Fear Conditioning and Associated Brain Regions After Pilocarpine-Induced Status Epilepticus

**DOI:** 10.3389/fnbeh.2020.00149

**Published:** 2020-08-27

**Authors:** Xiangmiao Qiu, Masako Kinoshita, Anjiao Peng, Wanling Li, Wanlin Lai, Jing Wang, Lin Zhang, Lei Chen

**Affiliations:** ^1^Neurology Department, West China Hospital, Sichuan University, Chengdu, China; ^2^Department of Neurology, National Hospital Organization, Utano National Hospital, Kyoto, Japan; ^3^Laboratory of Anesthesia and Critical Care Medicine, Translational Neuroscience Center, West China Hospital, Sichuan University, Chengdu, China

**Keywords:** status epilepticus, epilepsy, cognition, machine learning, animal models

## Abstract

Our study aimed to demonstrate time-dependent declarative memory changes and its associated brain regions after status epilepticus (SE) using structural imaging techniques and machine learning methods. Pilocarpine was administrated to establish the SE model. At four different time points after SE (1, 2, 3, and 4 months, respectively), rats were subjected to structural imaging acquisition as well as contextual fear conditioning for the measurement of brain structural changes and declarative memory. Voxel-based morphometry (VBM) analysis were performed. Those significantly different regions were selected as features for training support vector machine (SVM). A linear kernel was chosen for regression of declarative memory. Leave-one-out cross-validation was applied to ensure generalization. Our results showed that the pilocarpine groups displayed the most severely impaired declarative memory at 2 months after SE and improved afterward, but failed to recover to the normal condition at 4 months after SE. The pilocarpine groups showed lower gray matter volumes and larger cerebrospinal fluid (CSF) volumes. After controlling for the total brain volumes, ANOVA demonstrated gray matter volume changes in the CA1 subfield of the hippocampus, primary somatosensory cortex, entorhinal cortex, etc. The combination of VBM and SVM identifies the somatosensory cortex and entorhinal cortex as the correlated brain regions for declarative memory dysfunctions after SE. Our study indicates that compensational mechanisms might be triggered to help with the recovery of memory functions after SE. Structural changes of the somatosensory cortex and entorhinal cortex might be involved in memory impairment after SE.

## Introduction

Status epilepticus (SE), as an extreme form of epileptic seizures, can prolong for a sufficient length of time and produce irreversible insult to the brain (Trinka et al., 2015). Long-term sequelae of SE could ultimately devastate patients’ quality of life and impose an overwhelming social and economic burden (Sculier et al., 2018).

The current definition of SE designates a time point *t*_2_, after which long-term pathological changes, such as neuronal injury, neuronal death, and neuronal networks, were reported to occur. This time point *t*_2_ was proposed as an operational dimension to indicate when long-term, irreversible sequelae may appear. However, data regarding the clinical long-term sequelae of convulsive SE are yet incomplete. Specifically, long-term cognitive sequelae after SE remain debatable, with inconsistent results (Adachi et al., 2005; Power et al., 2018; Sculier et al., 2018). Long follow-up duration and tons of confounding factors might impose difficulties on clinical studies to reveal the impact of SE on cognition. Also, heterogenicity among human studies also makes it difficult to draw up a conclusion. Thus, in this study, we design an experimental study in order to complement evidence of long-term cognitive sequelae after convulsive SE.

Targeting declarative memory, we aim to demonstrate the time-dependent changes of contextual fear conditioning after SE in a rat pilocarpine-induced SE model. Moreover, using structural neuroimaging techniques and applying machine learning methods which are more sensitive, we also explore brain regions associated with cognitive deficits from the perspective of the whole brain.

## Materials and Methods

### Animals

A total of 45 male Wistar rats (8 weeks old, weight = 275–315 g) were purchased from Chengdu Dossy Experimental Animals Co., Ltd. All rats were group-housed (five per cage) in a 12-h dark/light cycle (8:00 p.m. to 8:00 a.m.) with food and water *ad libitum*. The pilocarpine-induced SE model, as a commonly used post-SE model, has a characteristic latent period (which is from days to weeks, according to previous reports) before spontaneous seizures, thus could more represent the most common variant of acquired human epilepsy. Given this advantage, we chose pilocarpine-induced SE model in our study. In post-SE animal models, the latent period usually lasts from days to weeks. Previous studies have reported that, in rodents, about 30 days post-SE, all animals have been observed to have spontaneous seizures (Pitkänen et al., 2017). As the same interval between different time points would make it easy to assess the changes with time, we chose 1 month as the interval in our study. Rats were randomly allocated to five groups: control group (*n* = 5), pilo-one group (*n* = 10), pilo-two group (*n* = 10), pilo-three group (*n* = 10), and pilo-four group (*n* = 10). Drug doses and administration were followed according to the protocols provided in Pitkänen et al. (2017). All 40 rats in the pilo groups were induced to SE at the same time by injection of a dose of pilocarpine (30 mg/kg) intraperitoneally. Pretreatment with a muscarinic antagonist intraperitoneally (atropine, 5 mg/kg) 30 min in advance was conducted to reduce the peripheral adverse effects (Pitkänen et al., 2017). Thirty minutes after reaching SE, diazepam (20 mg/kg, i.p.; #P-6503, Sigma) was given to terminate SE. Lithium (127 mg/kg; Sigma) was administrated intraperitoneally 24 h before the injection of pilocarpine to lower its dose (Pitkänen et al., 2017). Controls (*n* = 5) were given the same amount of atropine/lithium for pretreatment and pilocarpine was substituted by saline. Recurrent seizures were monitored *via* video recording without the use of electroencephalography (EEG).

This study was approved by the Animal Ethics Committee of West China Hospital (approval code: 2018113A). The experiments have been conducted according to the national guidelines about animal experiments, including ARRIVE guidelines and the UK Animals (Scientific Procedures) Act, 1986, and associated guidelines.

### Magnetic Resonance Imaging Acquisition

The control group was subjected to MRI scanning before the first injection of saline. Imaging acquisition of the pilo-one, pilo-two, pilo-three, and pilo-four groups were conducted respectively at 28, 61, 86, and 114 days after SE. Rats in the same group were scanned on the same day.

Magnetic resonance imaging was conducted in a 7T magnet (Bruker Biospec 70/30 USR, Ettlingen, Germany) using a surface coil (diameter, 72 mm). Rats were anesthetized with 2–4% isoflurane and 1.5 L/min O_2_. A bite-bar and a gas mask were used while the rats were placed in a prone position on an MRI bed. Breathing was monitored throughout the scan. The total acquisition time for each scan was about 5 min.

The MRI protocol included turbo rapid acquisition with relaxation enhancement sequence (RARE) T2-weighted with both coronal and transverse orientations. The imaging parameters for the coronal orientation were: repetition time (TR) = 4,202 ms; echo time (TE) = 33 ms; field of view (FOV) = 30 mm^2^ × 30 mm^2^, 40 slices; 0.5-mm slice thickness; and in-plane resolution of 0.117 mm^2^ × 0.117 mm^2^ (matrix, 256 × 256).

### Contextual Fear Conditioning

For all groups, contextual fear conditioning was conducted 3 or 4 days after imaging acquisition to assess the time-dependent declarative memory. Pilo-one group was assessed 1 month after SE and pilo-two 2 months, pilo-three 3 months, and pilo-four 4 months after SE. In order to keep the same odors during the whole contextual fear conditioning, before starting, we used 70% alcohol solution to wipe out the chamber. Then, each rat was placed in the illuminated chamber for 3 min to explore and take in the aspects of the chamber. Immediately after the 3-min habituation period, the rat was given foot shock for 2 s (0.8 mA). Fifteen seconds after the shock, we removed the rats from the chamber. The same solution was also used between animals. On the second day, the rats were placed back in the training chamber for 3 min to measure the freezing behavior. Both the training session and the testing session used the same solution. The freezing score, freezing time, and freezing episodes in the training and testing sessions were recorded by ANY-maze software.

Declarative memory represents a person’s ability to recollect past experiences. Patients with declarative memory impairment might have difficulty answering questions like, what did you eat for lunch yesterday? Or, where have you been? The impairment of this ability is quite often a complaint of epilepsy patients in clinical practice. In rodent animals, contextual fear learning studies the animals’ ability to pair the surroundings (the inner characteristics of the chamber) with the stimulus (the foot shock). When placed back in the same chamber, the recall of the surroundings might awaken fear memory in normal conditions. As the mechanisms of contextual fear conditioning were considered as the same ones that support declarative memory in humans (Rudy et al., 2004), contextual fear conditioning has been used as an excellent model for assessing declarative memory (Rudy et al., 2004). One of the biggest advantages of contextual fear conditioning is that it uses fear response as a measurement and, thus, minimizes the effect of motor deficits. As in contextual fear conditioning, repetitive foot shock might enhance declarative memory. To avoid this confounding, we did not use a longitudinal design in which each rat was followed up. In the present study, all rats were only administered foot shock once.

### Voxel-Based Morphometry Analysis

Voxel-based morphometry (VBM) analysis was performed using the Statistical Parametric Mapping 12 (SPM12; Wellcome Trust Centre for Neuroimaging, London, United Kingdom) toolbox in MATLAB 2013b (MathWorks, Natick, MA, United States). Firstly, raw 2dseq data were converted to NFITI format using Bru2nii software (Bruker2NIfTI v1.0.20180303: by Matthew Brett, Andrew Janke, Mikaël Naveau, Chris Rorden, Windows 64-bit), where images were resized by a factor of 10. As the subsequent segmentation requires images to be roughly aligned to avoid strange results, we used FMRIB’s Linear Image Registration Tool (FLIRT) (Jenkinson and Smith, 2001) in FMRIB Software Library (FSL 4.0)^1^ to align with a Wistar rat template (Valdes-Hernandez et al., 2011). Although, the brains of rats after SE showed significant morphological changes with broad individual variations.

The images were then segmented into three categories (white matter, gray matter, and cerebrospinal fluid) using old segment batch in SPM12. A modulation step was included to enable a comparison of the voxel-wise gray matter volume. The images were then smoothed with a 3-mm full width at half maximum (FWHM) Gaussian kernel. Get_total.m plugin for SPM12 was used to estimate the total intracranial volume by summing the probability maps of white matter, gray matter, and cerebrospinal fluid (CSF).

To reveal structural changes in gray matter/white matter due to SE, group differences were evaluated using ANOVA (SPM12); with and without total brain volumes were entered as covariates. Family error rate (FWE) was used for multiple comparisons. The statistical threshold was set at 0.05. Brain regions with significant differences (without total brain volumes entered as a covariate) were saved as a brain mask for the subsequent support vector regression. XjView toolbox^2^, which was modified for rat brain (the same template described above was used), was used for visualization.

### Support Vector Regression

In the present study, we selected a linear kernel support vector machine (SVM) for fear score regression. The formula for linear regression models could be represented as:

$$\hat{Y}=\sum_{j=1}^{p}{\hat{\beta }}_{j}\,X_{j}+{\hat{\beta }}_{0}$$

where $\hat{Y}$ represents the predictive value of the fear response and *X*_*j*_ is the value of the *j*th feature for the subjects. *F*-maps (gray matter, white matter, as well as both), without controlling for the total brain volumes obtained in the above analysis using SPM, were transformed into a binary brain mask and used as mask in the regression model for feature selection. Thus, all abnormal brain regions showing statistically significant differences were entered into the model. As the total brain volume could also be responsible for the fear response, we did not control for the total brain volume in this regression model. Three regression models were respectively conducted using gray matter only, white matter only, and both gray matter and white matter. The accuracy of the regression model was measured by mean square error.

A linear regression model allows the direct extraction of a weight for each of the support vectors, therefore allowing an evaluation of the different features’ contributions to the fear response. These weight vectors were used to generate a map of the contributing regions in our present study for visualization. In the context of support vector regression, the procedure consists of testing and training phases. Support vector regression would finally find a function which predicts the fear response best.

We used the LIBSVM toolbox (Chang and Lin, 2011) for MATLAB to perform this regression. We applied the leave-one-out cross-validation approach, which regards all observations but one subject from each group to train the regression model, to validate the performance of the regression. Permutation testing was conducted to evaluate the model. The number of permutation times was set at 1,000.

### Immunofluorescence

After the imaging and fear response tests, all the rats were anesthetized by injecting chloral hydrate (0.5 ml/100 g). Brains were dissected from the skull and post-fixed in 4% paraformaldehyde for 24 h. Four-micrometer coronal sections were obtained to perform immunofluorescence staining. Briefly, the sections were incubated with NeuN antibody (1:600, rabbit polyclonal IgG, ab104225, Abcam), a neuronal marker, or glial fibrillary acidic protein (GFAP) antibody (1:200, rabbit, #16385-1-AP, Proteintech), an astrocyte marker, overnight at 4°C. Next, the sections were incubated with goat anti-rabbit IgG (Alexa Fluor488) at 37°C for 45 min. Sections were examined using a fluorescence microscope.

### Statistical Analysis

Comparisons of the brain volumes, including gray matter, white matter, CSF, and total volumes, were conducted using Kruskal test with *post hoc* analysis [false discovery rate (FDR) corrected]. We employed ANOVA to assess differences in the fear response among groups. All statistical analyses were carried out and graphs were drawn on Python (version 3.7).

## Results

### Animal Model

Pilocarpine was used for SE animal model establishment. Respectively, four (for pilo-one group), five (for pilo-two group), six (for pilo-three group), and five (for pilo-four group) rats survived in the pilo groups and were used for later data analysis. Animals mainly died 1–3 days after the induction of SE. This mortality is in accordance with previous reports about this model (Pitkänen et al., 2017). Six days after SE, rats began to develop seizures. At 1 month after SE, the daily cumulative seizure frequency for the pilo groups (daily total seizure number/rat number) has reached 1.0. The total seizure number for each group was added up for later correlation analysis. Pilo-four group experienced the maximum number of seizures, positively related with the follow-up duration.

### Contextual Fear Conditioning

**FIGURE 1:**
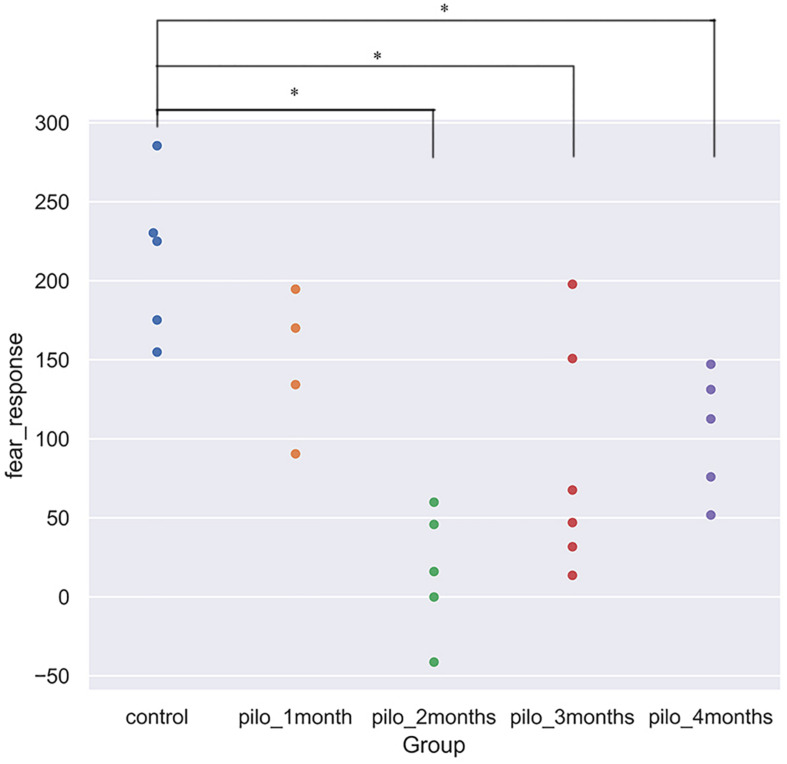
Fear response for each group (*P < 0.05, corrected). Control showed the best fear memory, while pilo-two group the worse. Fear response equals subtraction of the freezing score in the testing session from the freezing score in the training session. The higher the results, the better the declarative memory formed and retrieved. One rat in the pilo-two group got a negative score as it behaved actively and thus got a higher score in the testing session.

We detected significant changes on the freezing score in the training session, suggesting different baseline characteristics. Thus, we subtracted the freezing score in the testing session from the freezing score in the training session and regarded the results as fear response. The higher the subtraction results, the better the fear memory formed and retrieved. Significant differences on the fear response among groups were found using ANOVA (*P* < 0.001). After correction for multiple comparisons, control versus pilo-two (fear response decrease, 90.6%; *P* < 0.05), control versus pilo-three (fear response decrease, 60.4%; *P* < 0.05), and control versus pilo-four (fear response decrease, 51.6%; *P* < 0.05) still demonstrated significant differences, with the control groups demonstrating higher subtraction results, suggesting impaired fear memory formation and/or retrieval in the pilo groups. Figure 1 shows the fear response scores among groups. One of the animals had a negative fear response score as it did not freeze and behave more actively than the basic level and thus was given a higher score in the testing session. As we interpreted it as an inability to associate the aversive stimulus to the environment, we did not exclude it from the analysis. It appeared that dysfunction of fear memory demonstrated a time-dependent course. Fear memory was impaired 1 month after SE, and to the largest extent at 2 months. After that, fear memory improved, but failed to recover back to the normal level as the control group. No significant correlation was found between the total seizure number and fear response.

### Voxel-Based Morphometry

**FIGURE 2:**
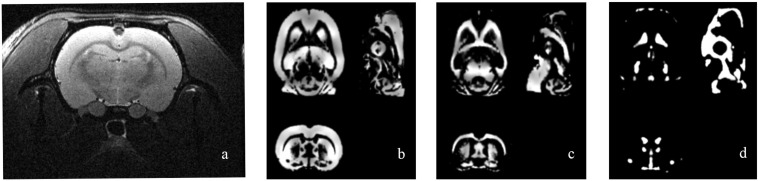
Structural image of one rat from the pilo-three group. **(a)** T2-weighted image before data processing. **(b–d)** Gray matter, white matter, and cerebrospinal fluid (CSF) images after voxel-based morphometry (VBM) segmentation.

**FIGURE 3:**
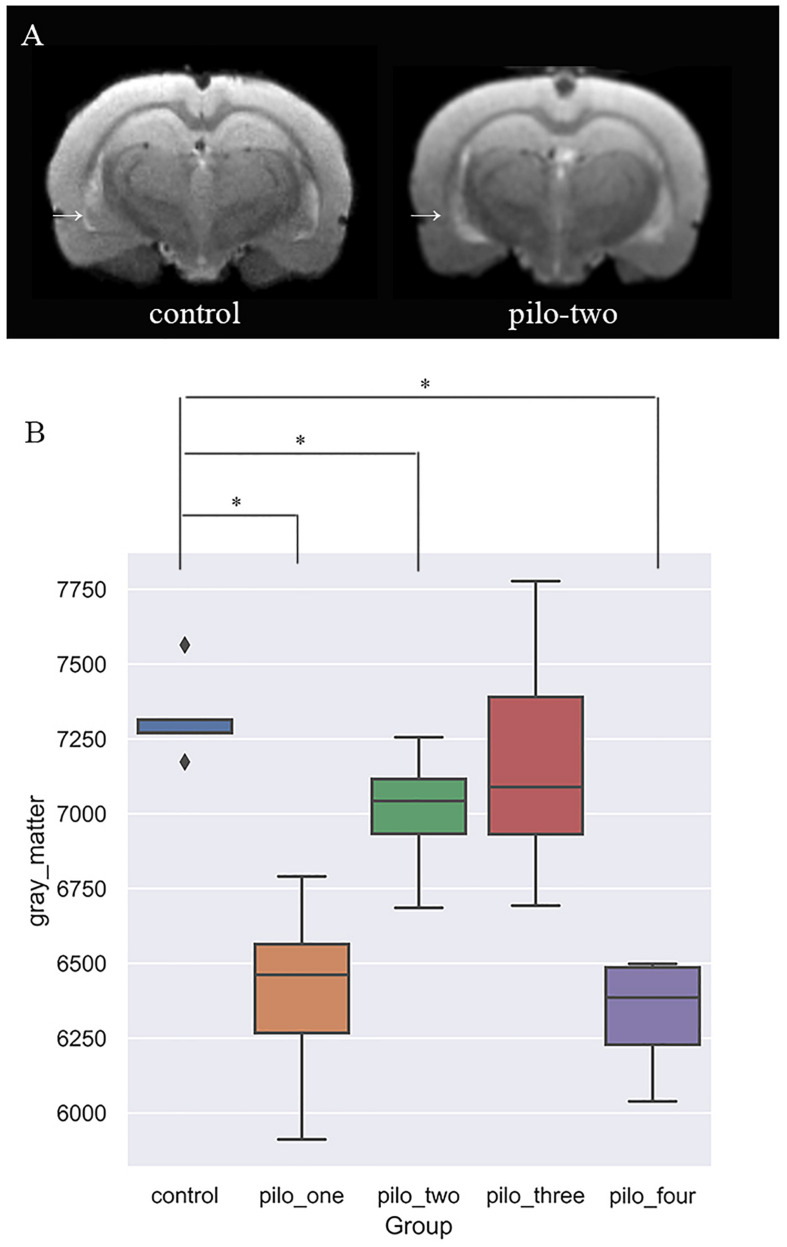
**(A)** T2-weighted image showing larger ventricular volumes after status epilepticus (SE). *Arrow*: larger lateral ventricular volume than the control. **(B)** Gray matter volumes decreased after SE, especially 1 month after SE (*P < 0.05).

Figure 2 shows the original T2 image as well as the gray matter, white matter, and cerebrospinal fluid images after segmentation. We first calculated the total gray matter volume of each rat and found statistically significant differences among groups (Kruskal–Wallis rank-sum test: *P* = 0.001). *Post hoc* analysis corrected by FDR revealed differences between the control versus pilo-one (*P* = 0.026), control versus pilo-two (*P* = 0.039), and control versus pilo-four (*P* = 0.026). Figure 3 shows the gray matter volumes of all five groups. Differences were also found among groups for total volume (*P* = 0.0114), white matter volume (*P* = 0.04481), and CSF volume (*P* = 0.00576). The control group had the smallest CSF volume, followed by pilo-one, pilo-four, and then pilo-two. Pilo-three group had the largest CSF volume.

In voxel-based morphometry, the *F*-maps for gray matter volume (GMV) after controlling for the total brain volumes were generated. Table 1 shows the significant differences with cluster size exceeding 50 voxels [*P* < 0.05, family wise error (FWE) corrected]. The most significantly affected regions were the bilateral ectorhinal and entorhinal cortices as expected. At the level of the cortex, the primary somatosensory cortex and the primary motor cortex were impacted. For the hippocampus, mainly the CA1 subfields were impacted. *Post hoc* analysis demonstrated decreased gray matter volumes in the CA1, the ectorhinal cortex, and the primary somatosensory cortex of pilo groups as compared to those of the control group.

**TABLE 1:** The most discriminant brain regions among groups.

Region	Peak intensity	Total voxels
Ect_right	37.165085	266
Ect_left	28.056425	198
DLEnt_left	28.461069	92
DLEnt_right	36.994576	183
S1BF_right	26.1962	436
S1BF_left	28.770287	376
Cg2_left	45.8676	142
M1_left	51.8117	516
RSD_left	24.0876	54
V1_left	57.2096	89
V2L_right	38.8489	54
S1ULp_left	29.1971	200
Cg2_right	34.2406	120

**Ect*, ectorhinal cortex; *DLEnt*, dorsolateral entorhinal cortex; *S1BF*, primary somatosensory cortex, barrel field; *M1*, primary motor cortex; *Cg2*, cingulate cortex, area 2; *V2L*, secondary visual cortex, lateral area; *RSD*, retrosplenial dysgranular cortex.*

### Support Vector Regression

**FIGURE 4:**
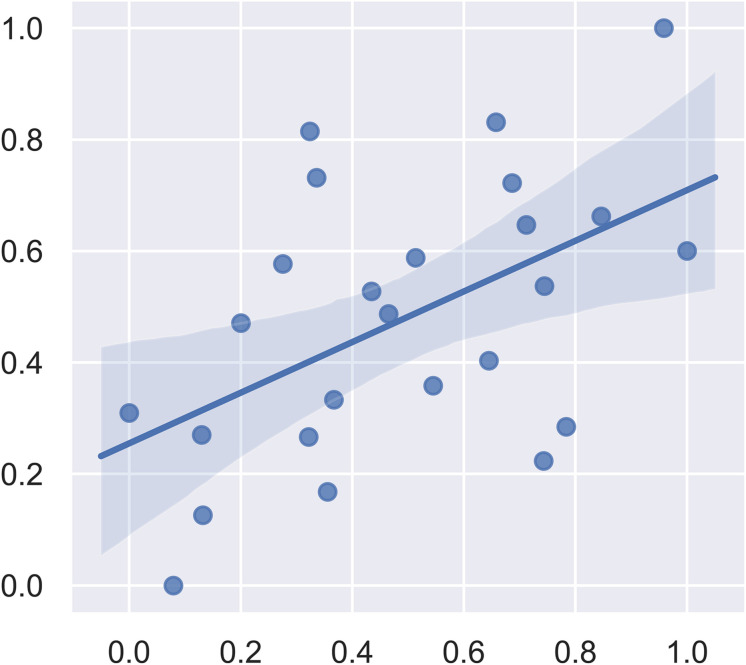
Plot of the actual fear response (scaled) and the predicted fear response (scaled) from the linear support vector regression model.

We first included all the brain regions in the SVM regression. In this model, the correlation between the predicted fear response and the actual fear response was 0.1049. The *P* value for the correlation from the permutation test was 0.63. Subsequently, we used *F*-maps for gray matter as a mask and included all abnormal brain regions that crossed the statistical threshold. The correlation between the predicted fear response and the actual fear response was 0.5175. The *P* value for the mean square error from the permutation test was 0.008. Figure 4 summarizes the results of the fear response regression model. We also applied white matter images and the combination of white matter and gray matter images for regression. The most satisfying accuracy was obtained using only gray matter images. The regression model using both gray matter and white matter images could not survive the permutation test. These results suggest that gray matter structural brain anomalies contributed more to the declarative memory dysfunction in post-SE than did whiter matter anomalies.

**FIGURE 5:**
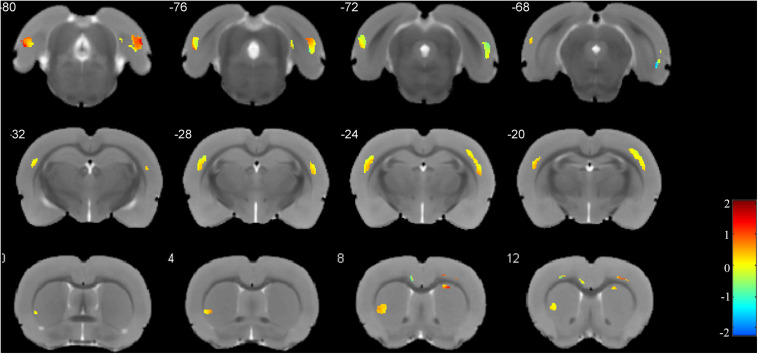
Weights for the different brain regions in the regression model. *Blue color scale* represents the negative weight vectors, while *red color scale* the positive weight vectors.

The weight vector maps showing a spatial pattern contributing to the freezing behavior are displayed in Figure 5. As shown, the primary somatosensory cortex, entorhinal cortex, and ectorhinal cortex carry the most contributing characteristics to declarative memory.

### Immunofluorescence

**FIGURE 6:**
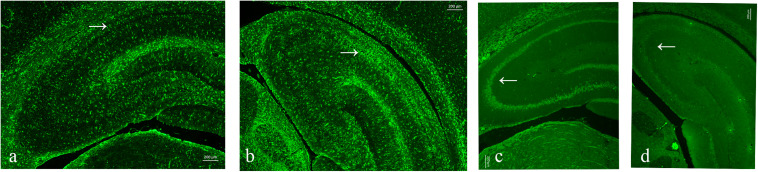
Neuronal loss and gliosis in the hippocampus with the impact of status epilepticus (SE). **(a,b)** Glial fibrillary acidic protein (GFAP) staining from the control **(a)** and pilo **(b)** groups. **(c,d)** NeuN staining from the control **(c)** and pilo **(d)** groups.

As neuronal loss and gliosis are the two main characteristics of hippocampal sclerosis in epilepsy, in order to evaluate the effect of pilocarpine-induced SE on the hippocampal neurons and astrocytes in our study, immunofluorescence was carried out against both NeuN and GFAP. Our data showed that there was a marked neuronal loss and gliosis in the SE groups compared to the control (Figure 6).

## Discussion

The present study has two main findings. Firstly, a time-dependent impairment of declarative memory after SE was demonstrated (first impaired and then improved, but failed to recover to the control level). Secondly, the combination of SVM and conventional VBM identified several extra-hippocampal brain regions possibly related to declarative memory impairment, including the primary somatosensory cortex and the entorhinal cortex.

Previous animal studies assessing declarative memory at only one time point also reported impaired fear conditioning 3 days (Lima et al., 2016), 2 months (Kemppainen et al., 2006; Zhang et al., 2010), and 3 months (Cardoso et al., 2009; Lesting et al., 2011) after SE. Controlling for experimental conditions, our study evaluated declarative memory at four different time points after SE, making it possible to demonstrate a time-dependent curve of memory impairment after SE. This inverted bell-shaped curve indicated that long-term memory sequelae happen shortly after SE, and at a certain time, some compensational mechanisms might be triggered to help with the recovery of memory functions. However, the memory function worse than the control at the ending measuring point suggests that having compensational mechanisms is not enough to recover back to the normal level. Potential intervention might be needed for the full recovery of memory function. Still, it has to be noted that there is a possibility that memory function could recover totally to the normal line with longer follow-up duration than that in our study.

Some factors such as medication, recurrent seizures, and aging might affect the performance of memory function. In our study, diazepam was prescribed only once to terminate SE. One dose of diazepam has indeed been shown in another animal study (Casasola-Castro et al., 2017) to impact on memory function in the long run. Moreover, a recent prospective SE patient cohort (Power et al., 2018), in which memory performance was also evaluated at different time points, proposed that improvement of cognitive function could be partially attributed to the declining medication effect. Thus, the possibility that failure to recover back to normal might be related to the medication could not be excluded. Our study design is unable to assess the effect of medication. An exact relationship between the usage of medication and cognition remains to be further investigated.

As for recurrent seizures, we did not find a significant association between spontaneous recurrent seizures and fear response. In our study, as the imaging data are rather high-dimensional and we have a very limited sample size, we chose an analysis strategy in which we do the association analysis (for seizure frequency and fear response) and ANOVA (for brain regions associated with SE) first to select relevant features before SVM. This analysis strategy could be more sensitive for detecting subtle differences. As a result, we did not include seizure frequency in the SVM analysis. Our negative study result is consistent with another animal study using a pilocarpine-induced SE model (Hort et al., 1999). Moreover, in another recent study (Pascente et al., 2016) using pilocarpine-induced SE, accelerated forgetting and a reduced learning rate were reported to be potential early biomarkers of epileptogenesis, suggesting that memory dysfunction, which occurred even before the spontaneous seizures, might not result from recurrent seizures. Yet, in clinical practice, there is one case report showing that patients’ cognitive function recovered 1 year after seizure remission (Van Paesschen et al., 2007). More evidence is needed to draw a conclusion. It has to be noted that cognitive function is a broad term covering various dimensions, with each having its own mechanisms and evaluation methods, resulting in the different cognitive tests and experimental paradigms used in different studies, thus adding more difficulties in clarifying the relationship between a specific cognitive function and recurrent seizures. As the implantation of electrodes might severely influence the quality of imaging acquisition, our study did not monitor the electrocorticography to assess the potential effect of EEG on cognitive function.

As repetitive foot shock might enhance the fear memory, our study did not use a longitudinal design for the purpose of increasing the sensitivity to detect fear response differences among groups. Our study design, in compromise, introduced age differences among groups as a confounding factor. As demonstrated by other studies (Draganski et al., 2011; Hamezah et al., 2017), brain volume might change with aging. Thus, the total gray matter/white matter/brain volume changes in our study should be interpreted as a result of both aging and SE rather than SE only. To eliminate the effect of this confounding factor, we controlled for total brain volume (sum of the gray matter, white matter, and CSF volumes) while we did the voxel-based morphometry analysis to evaluate the impact of SE on specific brain regions. However, as this factor might also affect memory, we did not control for total brain volume changes while we did the fear response regression analysis. Previous studies (Kemppainen et al., 2006; Fournier et al., 2013; Botterill et al., 2015) have also explored brain regions correlated with cognitive dysfunctions after SE. Most of them focused on the hippocampus and amygdala from the view of molecular and pathological mechanisms with *a priori* hypothesis based on existing knowledge. Our study, without the predefined hypothesis that specific brain regions are dominant in declarative memory, identified extra-hippocampus brain regions. To our knowledge, this is the first study to apply machine learning methods in epilepsy animal models to explore brain regions associated with declarative memory.

SVM has been shown to be sensitive in detecting spatially distributed brain regions. The combination of SVM and conventional VBM is especially helpful in recognizing intercorrelated brain regions (Ecker et al., 2010), which, in our case, are the primary somatosensory cortex and the entorhinal cortex. These results are consistent with the current understanding of fear conditioning. In a meta-analysis of human functional MRI studies (Fullana et al., 2016), the primary somatosensory cortex was identified as part of the “fear network” in charge of fear conditioning. The entorhinal cortex has also long been known to provide the main cortical source of input to the hippocampus and dentate gyrus as a way of learning and memory (van Groen, 2001). Furthermore, direct circuits from the entorhinal cortex to the hippocampal CA1 have recently been reported to be responsible for associated learning (Li et al., 2017). Thus, our results validate the combination of VBM and SVM as a sensitive method for detecting responsible brain regions for cognitive dysfunction after SE.

In a previous imaging study using a 4.7-T MR scanner and a one-time-point design (Niessen et al., 2005), the volumes of several brain regions including the entorhinal cortex and the hippocampus were found to decrease after pilocarpine-induced SE. Yet, only the hippocampus was found to be correlated with memory performance. In detail, this study measured spatial memory rather than declarative memory, as we did. The imaging analysis methods are also different between this study and ours. In contrast with the previous study, although the hippocampal volumes changed with the impact of SE and hippocampal injury in histology was also confirmed according to our study results, SVM did not identify the hippocampus as the responsible brain region for declarative memory. A possible explanation for this result might be associated with the methodology of VBM. VBM, as it has been named, identifies differences based on voxels. As the volume changes in the hippocampus induced by SE were less than 50 voxels, a highly sensitive methodology or a larger sample size might be needed to assess the role of the hippocampus on declarative memory. On the other hand, our study results still highlight the important role of extra-hippocampal regions as these regions demonstrated more severely changed volumes than did the hippocampus.

The methodological limitation of this research is that we did not use a longitudinal design in which memory tests were repetitively measured. As a repetitive fear condition protocol might largely consolidate the memory and induce confoundings, we thus abandoned the longitudinal design. Our study also has a limited sample size, which might be the reason for the hippocampus failing to be identified in the regression model. Yet, we have used a more sensitive analysis strategy (including the usage of SVM and selection of relevant features before SVM) in order to detect subtle differences. As a result, we identified the primary somatosensory cortex and the entorhinal cortex as correlated brain regions. Lastly, we did not monitor epileptic discharges, which may have also played a role in cognitive dysfunction after SE.

In conclusion, the present study demonstrates the time course of declarative memory dysfunction after SE and also identifies correlated extra-hippocampal regions. The phenomenon that memory dysfunction failed to return to the normal level suggests that early intervention might be needed to improve the cognitive outcome after SE. The combination of SVM and VBM identified the primary somatosensory cortex and the entorhinal cortex as the correlated brain regions for declarative memory impairment after SE.

## Data Availability Statement

The raw data supporting the conclusions of this article will be made available by the authors, without undue reservation.

## Ethics Statement

The animal study was reviewed and approved by Animal Ethics Committee of West China Hospital (approval code: 2018113A).

## Author Contributions

XQ, MK, AP, LZ, and LC contributed to the conceptualization and investigation. XQ, MK, AP, and WLi contributed to the methodology. XQ, WLi, JW, WLa, and AP worked on the animal models. XQ, WLi, WLa, AP, JW, and LC did the brain imaging and data analysis. XQ, JW, AP, LZ, and LC did the fear conditioning. XQ, MK, and LC contributed to the writing – original draft preparation, review, and editing. XQ and LC did the supervision. All authors contributed to the article and approved the submitted version.

## Conflict of Interest

The authors declare that the research was conducted in the absence of any commercial or financial relationships that could be construed as a potential conflict of interest.

## Footnotes

1 http://www.fmrib.ox.ac.uk/fsl/

2 http://www.alivelearn.net/xjview
